# Association of Vitamin D Status With Metabolic Syndrome and Its Individual Risk Factors: A Cross-Sectional Study

**DOI:** 10.7759/cureus.38344

**Published:** 2023-04-30

**Authors:** Monika Pathania, Minakshi Dhar, Arjun Kumar, Sarama Saha, Rashmi Malhotra

**Affiliations:** 1 Internal Medicine, All India Institute of Medical Sciences, Rishikesh, IND; 2 Biochemistry, All India Institute of Medical Sciences, Rishikesh, IND; 3 Anatomy, All India Institute of Medical Sciences, Rishikesh, IND

**Keywords:** lipid metabolism, obesity-related illnesses, dyslipidemia, metabolic syndrome (mets), vitamin-d deficiency

## Abstract

Background

Vitamin D deficiency has been associated with metabolic syndrome and its related outcomes, including type 2 diabetes mellitus, cardiovascular disease, myocardial infarction, and stroke. However, studies in Indian populations have provided conflicting results.

Methods

This single-center cross-sectional study was conducted in a tertiary care hospital in north India to determine the prevalence of vitamin D deficiency in patients with metabolic syndrome and to study the correlations of individual components of metabolic syndrome with 25 hydroxy vitamin D levels. The study included 235 patients aged between 30 to 70 years who met the criteria for metabolic syndrome. Patients with diabetes, hypothyroidism, Cushing's, and other disorders affecting vitamin D status, on supplements of vitamin D or anti-dyslipidaemic drugs were excluded. Information regarding socio-demographic characteristics, medical history, and anthropometric measurements were collected. Blood samples were collected to assess vitamin D levels.

Results

The prevalence of vitamin D deficiency (<20 ng/ml) was 76% among the study population. There was a significant negative correlation between vitamin D levels and diastolic blood pressure (Spearman's rho: -0.134, 95% CI: -0.82,-0.260, p=0.040), fasting blood glucose (Spearman's rho: -0.142, 95% CI: -0.101,-0.269, p=0.029), A weak correlation was also found between vitamin D3 levels and total cholesterol (Spearman's rho: -0.246, 95% CI: -0.119,-0.367, p<0.001), triglyceride levels (Spearman's rho: -0.246, 95% CI: -0.118,-0.370, p<0.001) and low-density lipoprotein (LDL) levels (Spearman's rho: -0.229, 95% CI: -0.102,-0.351, p<0.001).

Conclusion

The study findings suggest that vitamin D deficiency is prevalent among patients with metabolic syndrome in north India. There is a significant negative correlation between vitamin D levels and some components of metabolic syndrome. This highlights the need for further research to understand the underlying mechanisms and potential benefits of vitamin D supplementation in this population. Identification of high-risk individuals for hypovitaminosis D can aid in streamlining treatment guidelines and preventing unnecessary prescription of investigations in developing countries.

## Introduction

Vitamin D is a fat-soluble prohormone that in the past was exclusively associated with bone health; however, with increasing evidence of the presence of vitamin D receptors in various tissues like the kidney, pancreas, prostate, and the immune system, its role in health and disease has expanded and has been attributed to hormonal activity, including endocrine, autocrine and paracrine functions [1,2]. Vitamin D deficiency is widespread, but the role of vitamin D in metabolic syndrome is not fully elucidated due to the inconsistency of results and widespread differences in serum vitamin D levels across geographical and ethnic dissimilarities.

Metabolic syndrome encompasses a spectrum of disorders, including central obesity, atherogenic dyslipidemia, elevated blood pressure, elevated blood glucose, and proinflammatory states [3]. People with metabolic syndrome have an increased risk of type 2 diabetes mellitus, cardiovascular disease, myocardial infarction, and stroke and twice the risk of death from these causes compared with people without the syndrome [4]. A recent meta-analysis showed that the prevalence of metabolic syndrome in India is 30% and is more commonly seen among older adults (>60 years old), women, and the urban population [5]. According to the National Cholesterol Education Program Adult Treatment Panel III (NCEP ATP III) definition, metabolic syndrome is present if three or more of the following five criteria are met: waist circumference over 40 inches (men) or 35 inches (women), blood pressure over 130/85 mmHg, fasting triglyceride (TG) level over 150 mg/dl, fasting high-density lipoprotein (HDL) cholesterol level less than 40 mg/dl (men) or 50 mg/dl (women) and fasting blood sugar over 100 mg/dl [6].

The prevalence of vitamin D deficiency is an epidemic even in a tropical sunlight-rich country like India, but the figures vary across regions. A study of 1150 patients in Western India revealed the prevalence of vitamin D deficiency (<20 ng/ml) as 70% and slightly higher in females (76%) [7]. The prevalence was higher in northern India, with a study of 1346 subjects in Delhi showing a prevalence of 92% [8]. A study of the urban elderly population in South India revealed a prevalence of 56% [9], and a similar study in West Bengal revealed a prevalence of 53% [10]. 

Several studies in the Western population have reflected on the inverse relationship between metabolic syndrome with vitamin D deficiency. Studies on the Indian population have, however, provided conflicting results. In a cross-sectional study of 441 Indians with a mean age of 39.7±12.8 years, vitamin D insufficient status was not associated with metabolic syndrome or insulin resistance in Asian Indians of either sex [11]. In another study in non-obese individuals, decreased 25 hydroxy (OH) vitamin D levels were found to be associated with total abdominal fat deposits (TAAT) but not with anthropometric or biochemical parameters [12]. A cross-sectional study by Utmani et al. in 174 patients showed that the mean serum vitamin D levels among those with metabolic syndrome were lower (16.50+/-9.06 ng/ml) compared to those without the syndrome (20.75+/-10.29 ng/ml; p=0.004) [13].

Hypovitaminosis D appears to be a risk factor for components of the metabolic syndrome and its outcomes, as reflected by the studies from the West; the mechanisms, although, are unclear. Vitamin D metabolism is affected by ethnicity and dietary habits; hence it is difficult to extrapolate the findings of the Western population to the Asian population. A meta-analysis of 28 studies demonstrated that higher serum 25OH vitamin D levels were associated with a 55% reduction in diabetes, a 51% decreased risk of metabolic syndrome, and a 33% lower risk of cardiovascular disease (CVD) [14].

This is a single-center cross-sectional study conducted in a tertiary care hospital in north India to study the prevalence of vitamin D deficiency in patients with metabolic syndrome. It also aims to study the correlations of individual components of metabolic syndrome with 25 hydroxy vitamin D levels. With the high predicted prevalence of vitamin D deficiency in India and due to the cost burden of the investigation itself, identifying high-risk conditions having hypovitaminosis D can aid in streamlining treatment guidelines and preventing the unnecessary prescription of investigations in developing countries. 

## Materials and methods

This is a single-center cross-sectional study conducted at a tertiary care center in Uttarakhand state of northern India. The research was approved on 29/09/2017, letter number IM/RC109/2016/34, by the Institutional Ethics Committee of All India Institute of Medical Sciences, Rishikesh. The sample size was calculated assuming the prevalence of metabolic syndrome in north India as 30%, as reported by previous studies [5], the level of significance was set to 0.05, the power of the study to 80%, and the margin of error to 5%. The final calculated sample size was 240. 

All the patients presenting to the general medicine OPD were screened for inclusion in the study. Patients having metabolic syndrome as per the NCEP ATP III definition and within 30 to 70 years of age were included in the study. Patients with diabetes, hypothyroidism, Cushing's, and other disorders affecting vitamin D status, on supplements of vitamin D or anti-dyslipidemic drugs were excluded from the study. A total of 235 patients were included after the initial screening.

Information regarding socio-demographic characteristics, smoking and alcohol status, past medical history, and medical history of hypertension (self-reported as diagnosed by a doctor) was collected using a semi-structured questionnaire. Anthropometric measurements were recorded. Height, waist, and hip circumference were recorded per the guidelines of the National Institutes of Health, 1998. All measurements were taken by trained researchers. Body mass index (BMI) was calculated by the standard formula. Overweight was defined as a BMI range of 23-24.9 kg/m^2^ and obese as BMI≥25. For the biochemical profile, after an overnight fast, venous blood samples were collected by a trained technician. The tests included the estimation of the fasting lipid profile and glycosylated hemoglobin (HBA1c) estimated using enzymatic kits. The chemiluminescence immunoassay (CLIA) technique was used to estimate 25-hydroxyvitamin D for vitamin D status.

The data were entered in Excel sheets (Microsoft® Corp., Redmond, US) and analyzed on SPSS software version 21.0 (IBM, Inc., Armonk, US). All The variables were grouped by measures of central tendency and measures of dispersion. T-tests were used for bivariate analysis. Linear regression analysis was performed with Pearson's correlation coefficient, and linear regression equations were created.

## Results

A total of 235 patients were included in the study. The mean age of the subjects was 43.81±10.45 years. One hundred and twenty subjects (51.1%) were males, while 115 subjects (48.9%) were females. The mean BMI was 29.31±4.99, and the waist-hip ratio (WHR) was 0.964±0.06. The mean systolic blood pressure was 135.83±18.93 mm of Hg, and diastolic blood pressure was 84.59±10.57 mm of Hg. The mean vitamin D3 level in the study population was 19.14±20.44 ng/dl. Out of all participants, 87.70% had vitamin D levels below the normal cut-off of 30 ng/dl (see Table 1). Lipid profile was also analyzed for all the study subjects. The mean total cholesterol was 192.84±53.03 mg/dl, the mean triglyceride level was 167.94±92.11 mg/dl, the mean low-density lipoprotein (LDL) level was 121.10±36.20 mg/dl, while the mean HDL level was 43.89±10.98 mg/dl (see Table 1).

**Table 1:** Descriptive analysis of continuous variables SBP - systolic blood pressure; DBP - diastolic blood pressure; FBS - fasting blood sugar; 25OH vitamin D - 25 hydroxy vitamin D; TSH - thyroid stimulating hormone; LDL - low-density lipoprotein; HDL - high-density lipoprotein

Parameters [normal range]	N	Mean	Standard deviation
Age (years)	235	43.81	10.45
Height (cm)	235	160.36	9.96
Weight (kg)	235	75.42	14.55
BMI (kg/m^2^)	235	29.31	4.99
Waist circumference (cm)	235	100.29	12.57
Male	120	101.00	12.20
Female	115	99.60	13.00
Hip circumference (cm)	235	104.26	12.51
Waist-hip ratio	235	0.96	0.06
SBP (mm of Hg)	235	135.83	18.93
DBP (mm of Hg)	235	84.59	10.57
FBS [70-99 mg /dl]	235	113.85	40.38
25OH vitamine D [30-100 ng/ml]	235	19.14	20.44
TSH [0.35-5.5 mIU/ml]	235	2.30	1.63
Total cholesterol [125-200 mg/dl]	235	192.84	53.03
Triglycerides [50-150 mg/dl]	235	167.94	92.11
LDL [100-130 mg/dl]	235	121.10	36.20
HDL [40-60 mg/dl]	235	43.89	10.98

Bivariate correlation analysis was performed to assess correlations between vitamin D3 levels and all other parameters. A weak negative correlation was found between vitamin D3 levels and diastolic blood pressure (Spearman's rho: -0.134, 95% CI: -0.82,-0.260, p=0.040) and fasting blood sugar levels (Spearman's rho: -0.142, 95% CI: -0.101,-0.269, p=0.029). A weak correlation was also found between vitamin D3 levels and total cholesterol (Spearman's rho: -0.246, 95% CI: -0.119,-0.367, p<0.001), triglyceride levels (Spearman's rho: -0.246, 95% CI: -0.118,-0.370, p<0.001), and LDL levels (Spearman's rho: -0.229, 95% CI: -0.102,-0.351, p<0.001; Tables 2-3).

**Table 2:** Spearman's correlation analysis for 25OH vitamin D levels with other variables 25OH vitamin D - 25 hydroxy vitamin D (ng/ml); Tg - triglycerides (mg/dl); LDL - low-density lipoprotein (mg/dl); HDL - high-density lipoprotein (mg/dl)

	25OH vitamin D	Total cholesterol	Tg	LDL	HDL
25OH vitamin D	1.00	-0.246 (p=0.001)	-0.246 (p=0.001)	-0.229 (p=0.002)	-0.075 (p=0.254)
Total cholesterol	-0.246 (p=0.001)	1.00	0.502 (p=0.000)	0.907 (p=0.000)	0.378 (p=0.000)
Tg	-0.246 (p=0.001)	0.502 (p=0.000)	1.00	0.528 (p=0.000)	-0.102 (p=0.118)
LDL	-0.229 (p=0.002)	0.907 (p=0.000)	0.528 (p=0.000)	1.00	0.237 (p=0.000)
HDL	-0.075 (p=0.254)	0.378 (p=0.000)	-0.102 (p=0.118)	0.237 (p=0.000)	1.00

**Table 3:** Spearman's correlation analysis for 25OH vitamin D levels with diastolic blood pressure and fasting blood sugar 25OH vitamin D - 25 hydroxy vitamin D (ng/ml); DBP - diastolic blood pressure (mm of Hg); FBS - fasting blood sugar (mg/dl)

	25OH vitamin D	DBP	FBS
25OH vitamin D	1.00	-0.134 (p=0.040)	-0.142 (p=0.029)
DBP	-0.134 (p=0.040)	1.00	0.132 (p=0.043)
FBS	-0.142 (p=0.029)	0.132 (p=0.043)	1.00

**Figure 1:**
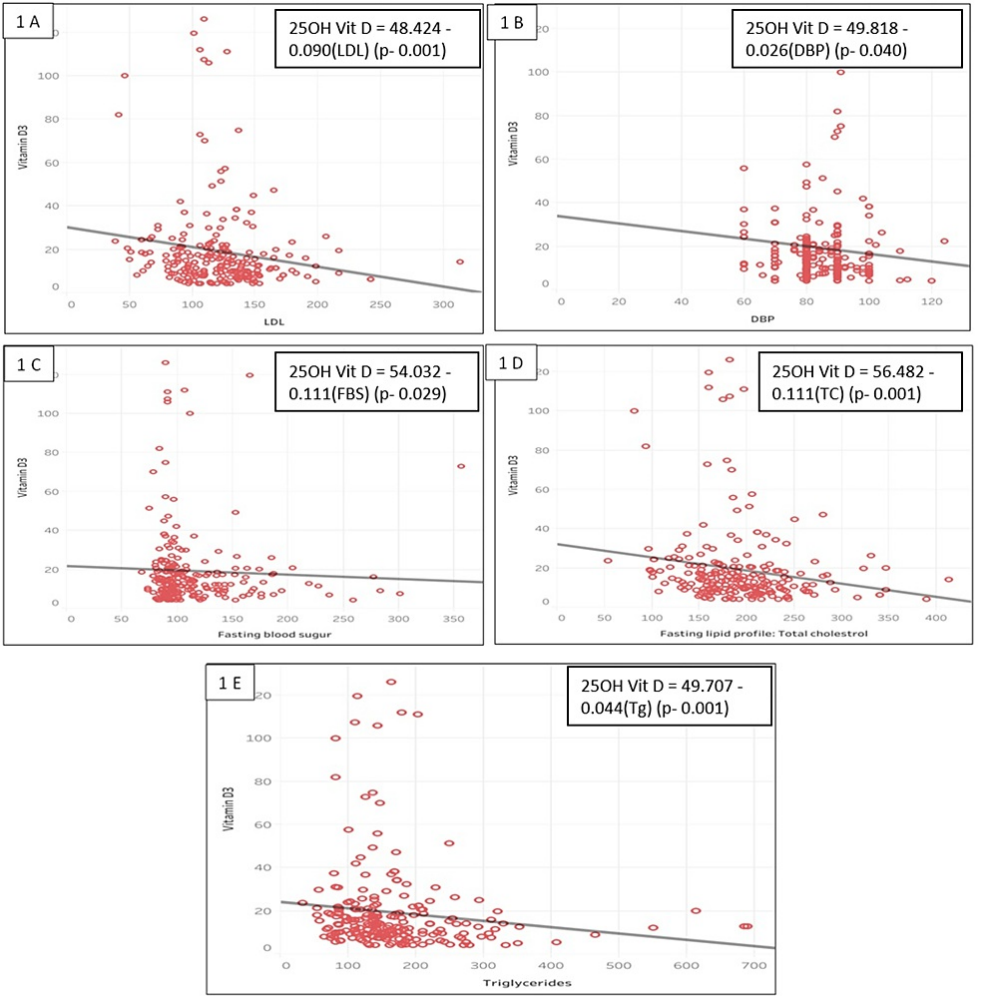
Images A-F depicting the negative correlation of 25OH vitamin D levels (ng/ml) with other parameters (LDL, DBP, FBS, TC, and Tg) 25OH vitamin D - 25 hydroxy vitamin D; DBP - diastolic blood pressure; FBS - fasting blood pressure; LDL - low-density lipoprotein; Tg - triglycerides; TC - total cholesterol

Regression equations were estimated, revealing that for every one-unit increase in diastolic blood pressure, fasting blood sugar, LDL cholesterol, triglycerides, or total cholesterol, vitamin D3 is estimated to decrease by 0.026, 0.111, 0.090, 0.044, or 0.111, respectively, holding other factors constant (see Table 4 and Figure 1).

**Table 4:** The estimated associations between various predictor variables (DBP, FBS, LDL, Tg, and TC) and 25OH vitamin D levels The regression equations provide an estimate of the amount of change in vitamin D3 for every one-unit increase in the predictor variable, assuming that all other factors remain constant. The associations are either negative or positive, indicating the direction of the relationship between the predictor variable and 25OH vitamin D. 25OH vitamin D - 25 hydroxy vitamin D; DBP - diastolic blood pressure; FBS - fasting blood pressure; LDL - low-density lipoprotein; Tg - triglycerides; TC - total cholesterol

Predictor variable	Regression equation	Estimated association
DBP	25OH vitamint D = 49.818 - 0.026(DBP)	Negative: -0.026
FBS	25OH vitamint D = 54.032 - 0.111(FBS)	Negative: -0.111
LDL	25OH vitamint D = 48.424 - 0.090(LDL)	Negative: -0.090
Tg	25OH vitamint D = 49.707 - 0.044(Tg)	Negative: -0.044
TC	25OH vitamint D = 56.482 - 0.111(TC)	Negative: -0.111

## Discussion

The prevalence of vitamin D insufficiency in our study population, keeping a cut-off of 30ng/ml, was 87.7%, and that of vitamin D deficiency with a cut-off of 20 ng/ml was 76%, with a mean value of 19.148 ng/ml. This is higher than the prevalence of vitamin D deficiency in the general population in India [7].The mean values were higher for females as compared to males, although the results were not significant. Gender differences in vitamin D deficiency have been variable across study populations in other studies [15,16]. In this study, a weak negative correlation was also found between vitamin D levels and diastolic blood pressure, and fasting blood sugar. A weak negative correlation was also seen between vitamin D levels and lipid profile parameters like total cholesterol, triglycerides, and LDL, but not HDL.

Although vitamin D is a liposoluble vitamin obtained through sunlight exposure, several factors like aging, skin pigmentation, obesity, and vitamin D intake influence it. It is now widely recognized that sunlight exposure leading to pre-vitamin D3 production is minimal above 35-degree latitude [17]. A high prevalence of vitamin D insufficiency in a sunlight-rich tropical country of India might appear counter-intuitive at first, but changing lifestyle and food habits, sedentary nature, reduced sunlight exposure with clothing and sunscreens, and increasing UVB absorbing pollutants can explain this conundrum [18]. 

A meta-analysis of 23 cross-sectional and cohort studies in the West showed that the prevalence of vitamin D deficiency was 35% higher in obese subjects compared to the eutrophic group (PR: 1.35; 95% CI: 1.21-1.50) and 24% higher than in the overweight group (PR: 1.24; 95% CI: 1.14-1.34) [19]. The principle mechanism agreed upon for the higher prevalence of vitamin D insufficiency and deficiency in obese individuals has been termed as volumetric dilution of vitamin D, which explains the higher distribution of vitamin D in obese individuals, lowering the serum concentrations of vitamin D [20]. Non-alcoholic fatty liver disease (NAFLD) is common in patients with metabolic syndrome, and this may result in impaired hepatic 25-hydroxylation [21]. Another study highlights the decreased expression of cytochrome P450 2J2 gene coding for the enzyme 25-hydroxylase and cytochrome P450 27B1 coding for the enzyme 1a-hydroxylase in obese individuals [22].

Vitamin D induces the activation of calpain and caspase-12, the enzymes involved in the apoptosis of fat tissue [23]. Blumberg et al. found that liganded vitamin D receptors of 3T3-L1 cells in the adipose tissue repressed both CCAAT/enhancer binding protein (C/EBP)-α and peroxisome proliferator-activated receptors (PPAR)-gammaexpression via inhibition of C/EBP-β expression and action and was a potent inhibitor of adipogenesis [24]. All these findings strengthen the hypothesis of the presence of vitamin D deficiency in metabolic syndrome patients, but the direction of causality is still debated.

The acceleration of atherosclerosis in obesity and metabolic syndrome has been well defined, but peripheral vascular disease, hypertension, and cardiac-associated mortality, in general, are all highly prevalent in vitamin D-deficient individuals, thus compounding the ill effects of obesity [25]. Despite the prevalence of both conditions, interventional trials with supplementation of vitamin D in patients at risk of or with established cardiovascular disease have not produced positive results [26].

Another finding in the study is a negative correlation of fasting lipid profile with vitamin D levels. A similar result was seen in a cross-sectional study of 3788 adults in China, showing a significant inverse correlation between 25(OH) vitamin D and triglycerides (β coefficient=-0.077, p<0.05) and LDL cholesterol (β coefficient =-0.245, p<0.05) [27]. A meta-analysis of 17 cross-sectional studies, including 25,394 subjects, found an inverse association between 25(OH) vitamin D and triglycerides, total cholesterol, and LDL cholesterol and a direct association with HDL cholesterol in children and adolescents [28]. A positive correlation with HDL was, however, not seen in our study. The mechanisms by which vitamin D could affect lipid profiles are not well defined. Vitamin D can affect lipoprotein metabolism and reduce triglyceride synthesis and secretion in the liver, increasing very low-density lipoprotein (VLDC) receptor expression. Hence its deficiency can lead to increased TG and VLDL levels [29]. Suggested mechanisms also include vitamin-D-mediated suppression of parathyroid hormone secretion, intestinal absorption of calcium, and modulation of beta-cell function and insulin resistance [30]. 

Our study highlights some important points. Firstly, it provides evidence of a higher prevalence of vitamin D deficiency in metabolic syndrome and produces data reproducible for the Indian population. Secondly, it suggests the presence of higher diastolic blood pressure, fasting blood sugars, and dyslipidemia in this study population which has not been proven in the Indian population previously. However, there are also certain key limitations. Being a cross-sectional study, causality can not be established, and hence, prospective studies are needed to establish the direction of vitamin D deficiency and its role in producing metabolic syndrome or vice versa. Secondly, due to a lack of a control group in the study, a higher prevalence of vitamin D deficiency in this population can be suggested but not established with certainty. 

## Conclusions

To conclude, vitamin D deficiency is an accelerating health concern in the Indian population, and a higher prevalence of vitamin D deficiency is seen in the population with metabolic syndrome. Vitamin D deficiency in this population was found to be correlated with increased diastolic blood pressure and decreased total cholesterol, low-density lipoproteins, triglycerides, and fasting blood sugars. These findings suggest the potential advantage of adding vitamin D supplementation in this population group, although prospective studies are needed for their confirmation. 
